# Novel Strategy to Prevent Otitis Media Caused by Colonizing Streptococcus pneumoniae

**DOI:** 10.1371/journal.ppat.0030028

**Published:** 2007-03-23

**Authors:** Jonathan A McCullers, Åsa Karlström, Amy R Iverson, Jutta M Loeffler, Vincent A Fischetti

**Affiliations:** The Scripps Research Institute, United States of America

In early childhood, 70%–83% of children experience at least one episode of acute otitis media (AOM) [1,2]. Streptococcus pneumoniae is the most common bacterial agent identified as the causative agent of these infections, although there is increasing evidence that a variety of respiratory viruses play a prominent role in the development and pathogenesis of AOM [1]. Even though the heptavalent pneumococcal conjugate vaccine appears to be making an impact on the incidence of this disease among children in the United States, AOM (with more than 24 million diagnoses annually) remains the leading reason for physician visits and antibiotic prescriptions among preschool-aged children [2,3]. Frequent use of antibiotics for AOM has led to a vicious cycle of diminishing returns: increased exposure has led to increasing drug resistance, which in turn makes the infections more difficult to treat, necessitating new drugs and more treatment. Recently, purified bacteriophage (phage) cell wall hydrolases, or lysins, have shown promise as novel anti-infectives due to their ability to eradicate nasal carriage of gram-positive pathogens, particularly S. pneumoniae [4,5]. These highly active enzymes are produced by phages to disrupt the bacterial cell wall for the release of progeny phage. Here, we show that the Cpl-1 lysin, which is specific for S. pneumoniae [6], prevents AOM in a novel mouse model that mimics the natural pathogenesis of this common infection.

Current animal models for AOM have critical limitations. Modeling AOM in mice requires invasive and artificial procedures to establish infection, and sacrifice of the animals to determine outcomes. Larger animals such as chinchillas and ferrets may develop infection by more natural routes, but use of these models is limited by their size and complexity [7,8]. Ideally, we wished to develop a non-invasive mouse model that was permissive of natural infection. We engineered a piliated strain of *S. pneumoniae,* known to efficiently colonize mucosal surfaces (a type 19F strain obtained from B. Henriques-Normark, ST162^19F^) [9], to express luciferase [10]. Groups of five mice maintained in a BL2 facility were infected intranasally with 1 × 10^5^ or 1 × 10^6^ colony-forming units (CFU) of this bioluminescent strain under light anesthesia with 2.5% inhaled isoflurane using an established infection model approved by the St. Jude Children's Research Hospital animal care and use committee [11]. Animals were followed daily for development of infection for two weeks and thrice weekly for another four weeks. Within 72 hours of pneumococcal infection, 100% of mice (10/10) were visibly colonized with bacteria in the anterior portion of their nose, and 70% (7/10) had developed AOM. These infections of the middle ear all resolved by bioluminescent imaging within 48 hours, and no mice had evidence of AOM six days after challenge or later. Nasal colonization persisted for a median of 27 days (range 17–34 days).

Around half of all children are colonized with S. pneumoniae [12]. Alteration of eustachian tube function or disruption of mucosal surfaces through viral infection allows colonizing bacteria to ascend into the middle ear, triggering AOM [1]. To model this phenomenon, we infected mice that had been stably colonized by pneumococcus with influenza virus and followed them for development of AOM. Although all mice had been colonized prior to infection with virus, 63% of virus-infected mice (19/30) developed AOM compared to 0% (0/10) of mice mock-infected with phosphate buffered saline (PBS) (Figure 1). Twenty-one of thirty mice in the virus group had experienced AOM after introduction of the bacteria in the first 72 hours post colonization (with resolution before viral challenge), while eight of ten mice in the PBS control group had experienced AOM with resolution (unpublished data). Both de novo and recurrent infections were seen in the virus-infected mice, with no correlation to whether they had previously had AOM. This is the first mouse model of AOM in which infection develops in a manner analogous to that observed in children.

**Figure 1:**
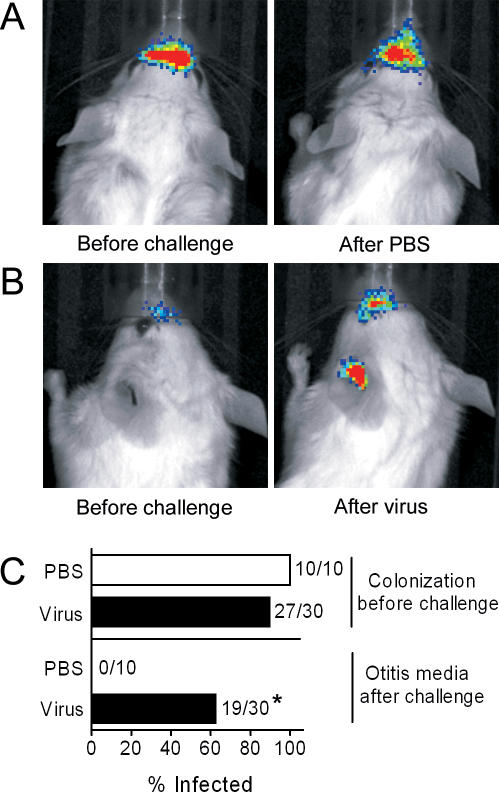
Visualization of Bioluminescent Bacteria Inside Live, Anesthetized Animals Shows the Induction of Otitis Media (A–B) Six- to eight-week-old female Balb/cJ mice (Jackson Laboratory, http://www.jax.org) were colonized intranasally with 1 × 10^5^ CFU of type 19F S. pneumoniae strain ST162^19F^ suspended in 100 μl of sterile PBS, then seven days later either (A) infected intranasally in a volume of 100 μl of sterile PBS with 100 TCID_50_ (doses of virus infectious for 50% of tissue culture wells) of the Mount Sinai strain of influenza virus A/Puerto Rico/8/34 (H1N1), or (B) mock-infected with sterile PBS. Mice were imaged for 60 seconds daily using an IVIS Lumina Imaging System (Xenogen, http://www.xenogen.com), and images were analyzed using Living Image software (version 2.50.1, Xenogen). (A) Pneumococcal load in the nose of a representative mouse colonized with pneumococcus remained the same after mock-infection with PBS, while (B) a representative mouse infected with influenza virus developed otitis media in the left ear. (C) Although colonization of mice on day 7 did not differ between the groups (*p* = 0.56), significantly more mice infected with influenza developed otitis media than did mock-infected animals (*p* = 0.00053). An asterisk indicates a significant difference between the groups using a 2-tailed Fisher's Exact test.

Using this novel and powerful model, we sought to test our hypothesis that reduction or elimination of colonizing pneumococci with purified Cpl-1 lysin [6] would prevent the development of AOM. Prior to infection with influenza virus, mice colonized with pneumococcus for seven days were treated twice four hours apart with either 1,000 ug of Cpl-1 intranasally or enzyme buffer (mock treatment). At the time of the second treatment, nine of ten animals (90%) had cleared the pneumococcus from the nose, compared to zero of ten (0%) treated with enzyme buffer. The Cpl-1 lysin was 100% effective in preventing AOM, as no animal treated with lysin developed a secondary bacterial infection following influenza infection, while eight of ten (80%) mice that were mock-treated developed AOM (Figure 2). In the one mouse in which colonization persisted despite lysin treatment, the amount of bacteria present decreased dramatically (78% reduction in flux of light through the nose). In addition, no mice treated with Cpl-1 and observed for the duration of these experiments developed toxicity or illness attributable to the enzyme as determined by clinical observation, weight loss, and histopathology (unpublished data).

**Figure 2:**
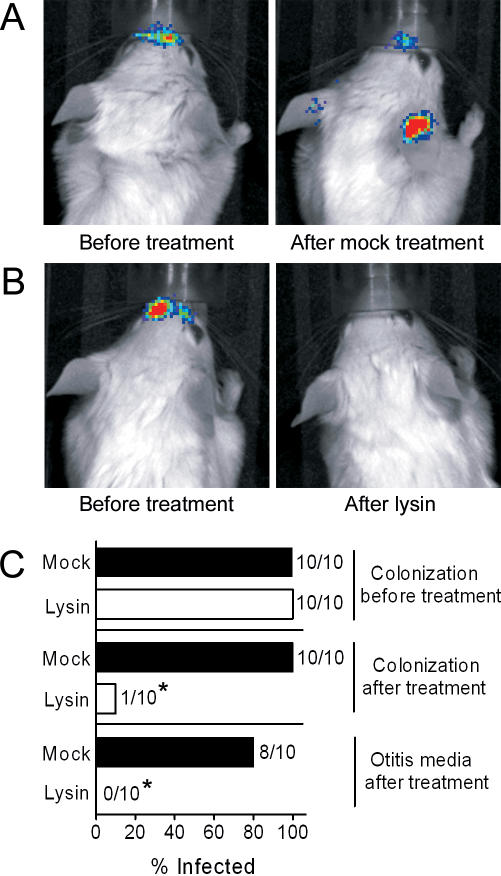
Treatment with Lysin Eliminates Colonization and Prevents the Development of Otitis Media (A–B) Mice were colonized intranasally with 1 × 10^5^ CFU of S. pneumoniae and seven days later were infected with influenza virus. Four hours prior to viral infection, and then again at the time of viral infection, mice were either (A) treated intranasally with 1,000 μg of Cpl-1 lysin suspended in 20 μl of enzyme buffer^4^, or (B) mock treated with 20 μl of enzyme buffer. Mice were imaged for 60 seconds daily. (A) A representative mouse mock-treated with enzyme buffer develops bilateral otitis media 24 hours after infection with influenza virus, while (B) a representative mouse treated with lysin clears the colonizing pneumococci and does not develop a secondary bacterial infection. (C) Although colonization of mice on day 7 did not differ between the groups (*p* = 1.00), significantly fewer mice treated with Cpl-1 remained colonized 24 hours later (*p* = 0.00012), and no treated mice developed otitis media compared to mock-treated animals, in which otitis media was seen in 80% (*p* = 0.00036). An asterisk indicates a significant difference between the groups using a 2-tailed Fisher's Exact test.

This novel model of naturally developing AOM will be useful in studies of prevention and treatment of this important and common infection. Our data on the use of lysin in the model suggest that a strategy of decolonizing children of S. pneumoniae may prevent many cases of AOM, particularly those with chronic AOM. Importantly, these data also suggest that elimination or even reduction of resident pathogenic bacteria can prevent secondary bacterial complications of influenza. In support of this view, a recent study of a pediatric pneumococcal vaccine in South Africa showed a 31% decrease in the incidence of virus-associated pneumonia compared to controls [13]. Secondary bacterial infections account for much of the morbidity and approximately 25% of all deaths during seasonal epidemics of influenza, as well as 50%–95% of deaths during pandemics of influenza [14]. It is very likely that a reduction in pneumococcal colonization in susceptible populations such as infants and the elderly during a pandemic or annual influenza outbreak could result in a concomitant reduction in morbidity and mortality. With worldwide concerns over a potentially incipient pandemic with highly pathogenic influenza viruses of the H5N1 subtype, further study of novel therapeutics such as phage-derived lysins to prevent these infections is warranted. 

## Abbreviations

AOM: acute otitis media
PBS: phosphate buffered saline
